# Designing Lightweight 3D-Printable Bioinspired Structures for Enhanced Compression and Energy Absorption Properties

**DOI:** 10.3390/polym16060729

**Published:** 2024-03-07

**Authors:** Akhil Harish, Naser A. Alsaleh, Mahmoud Ahmadein, Abdullah A. Elfar, Joy Djuansjah, Hany Hassanin, Mahmoud Ahmed El-Sayed, Khamis Essa

**Affiliations:** 1 Department of Mechanical Engineering, University of Birmingham, Edgbaston, Birmingham B15 2TT, UK; 2 Department of Mechanical Engineering, Imam Mohammad Ibn Saud Islamic University (IMSIU), Riyadh 11432, Saudi Arabia; naalsaleh@imamu.edu.sa (N.A.A.); aaelfar@imamu.edu.sa (A.A.E.); jrdjuansjah@imamu.edu.sa (J.D.); 3 Department of Production Engineering and Mechanical Design, Tanta University, Tanta 31512, Egypt; m.ahmadein@f-eng.tanta.edu.eg; 4 Faculty of Engineering, Helwan University, Cairo 11795, Egypt; 5 School of Engineering, Technology, and Design, Canterbury Christ Church University, Canterbury B15 2TT, UK; 6 Department of Industrial and Management Engineering, Arab Academy for Science Technology and Maritime Transport, Alexandria 21599, Egypt; dr.mahmoudelsayed12@gmail.com

**Keywords:** additive manufacturing, bio-inspired design, lattice structure, energy absorption, light-weight aerospace structures

## Abstract

Recent progress in additive manufacturing, also known as 3D printing, has offered several benefits, including high geometrical freedom and the ability to create bioinspired structures with intricate details. Mantis shrimp can scrape the shells of prey molluscs with its hammer-shaped stick, while beetles have highly adapted forewings that are lightweight, tough, and strong. This paper introduces a design approach for bioinspired lattice structures by mimicking the internal microstructures of a beetle’s forewing, a mantis shrimp’s shell, and a mantis shrimp’s dactyl club, with improved mechanical properties. Finite element analysis (FEA) and experimental characterisation of 3D printed polylactic acid (PLA) samples with bioinspired structures were performed to determine their compression and impact properties. The results showed that designing a bioinspired lattice with unit cells parallel to the load direction improved quasi-static compressive performance, among other lattice structures. The gyroid honeycomb lattice design of the insect forewings and mantis shrimp dactyl clubs outperformed the gyroid honeycomb design of the mantis shrimp shell, with improvements in ultimate mechanical strength, Young’s modulus, and drop weight impact. On the other hand, hybrid designs created by merging two different designs reduced bending deformation to control collapse during drop weight impact. This work holds promise for the development of bioinspired lattices employing designs with improved properties, which can have potential implications for lightweight high-performance applications.

## 1. Introduction

The demand for lightweight structures with improved strength and energy absorption properties is exponentially growing in multiple applications, spanning biomedical, aerospace, and defence. Addressing this need, studies have been emerging in developing lightweight structures with improved properties, such as shafts [1,2], sandwich panels [3,4], plates [5], foams, and honeycomb structures [6,7,8]. While these structures have shown great promise in terms of their mechanical properties, there is still room for optimisation. To improve the mechanical properties and energy absorption capability of lightweight structures, bio-inspired lattice structures have been explored to offer an exceptional combination of mass reduction, high Young’s modulus, ultimate strength, and enhanced energy absorption [9,10]. To achieve improved mechanical properties, a comprehensive understanding of the structural geometrical parameters of the designed nature-inspired lattice structures is essential. This includes cell size, relative density, and topology, and their impact on mechanical behaviour. Recent advancements in additive manufacturing (AM) technology have transformed the capacity to design and produce intricate structures with improved precision [11,12,13]. These technological advancements have motivated researchers to explore bioinspired lattice designs and materials spanning a wide range of engineering applications [14,15,16,17]. As a result, new horizons have opened in the pursuit of lightweight, high-performance components that show superior mechanical and energy absorption properties.

Fused deposition modelling (FDM), a widely used AM technology introduced by Scott Crump in the late 1980s [18] offers a range of benefits. These include simplicity, environmental friendliness, and a well-established ecosystem of materials and hardware [19]. In the FDM process, a spool of thermoplastic filament is fed through a tube into the 3D printer. This filament, typically a thermoplastic material, is then guided through an extrusion head equipped with a heated nozzle where it is melted. The printer deposits the softened material layer by layer and shapes it each into a thin cross-section according to a digital design. Each layer is cooled and solidified before the FDM process continues until the final part is fully printed.

The elastoplastic behaviour of different engineering structures has been the subject of hundreds of earlier studies. Due to the complicated and nonlinear relationship between stress and strain, numerical techniques are often applied to analyse such complex problems for various engineering applications. These methods include the finite element method (FEM), which is the most widely used numerical method because of its flexibility in handling material nonlinearity and heterogeneity [20]. It discretises the domain into finite elements to approximate the behaviour of structures under different loading conditions. However, other techniques also exist, such as the Finite Difference Method, which is a robust meshless method that uses Taylor series expansions to derive explicit formulae for the required partial derivatives of unknown variables [21]. In addition, the Bézier Multi-Step Method [22] was shown to be a computationally efficient technique for solving the governing nonlinear equation using Bézier curves.

Nature offers many architectures that are lightweight yet have outstanding mechanical and high energy-absorbing properties. Specific fruit shells and rinds have layered designs to absorb impact. The coconut husk, for example, can absorb more than 100 joules through deformation and cracking. Its inner shell makes it tough through its laminated structure. The macadamia nut shell can absorb energy by plastic deformation, allowing it to buckle and compress [23]. The spongy mesocarp of some fruits, like the pomelo, offers cushioning by deforming cell walls, flowing fluid, and cell buckling. Durian’s spikes improve interactions with branches during falls to dissipate impact energy [24]. Spider silk and abalone nacre also exhibit efficient energy-dissipating microstructures. Spider silk absorbs impact energy by unravelling its nanocrystals, while nacre utilises its layered platelet structure to improve its toughness [25]. Organisms also employ energy-absorbing adaptations, such as in the helicoidal fibres of mantis shrimp clubs, to localise damage. Cow and sheep horns act as crumple zones by buckling pillars [8]. Armoured fish scales distribute penetrating impacts [26].

The literature on mantis shrimp dactyl clubs has focused on their failure mechanisms and their capability to absorb impact energy [27,28]. Weaver et al. [29] studied the microstructural characteristics of shrimp dactyl clubs, including their Young’s modulus, graded microstructure, and hardness of the outer layer. These characteristics were found to efficiently reduce structural failure when the dactyl clubs were subjected to uniaxial loading. In particular, the elastic modulus of the herringbone fibre was found to outperform the helicoidal fibre design. Additionally, the herringbone fibre architecture showed enhanced toughness by widening the fracture propagation channel, thereby increasing energy absorption. In another study, Amini et al. [30] studied the quasi-plastic bending of dactyl club impact. They reported that the lower structural layer showed strain hardening and increased energy absorption due to microchannel densification. Chua et al. [31] demonstrated that the crack-tip plastic energy dissipation was due to the dactyl club fracture toughness strengthening stiffness. This was in agreement with Taylor and Patek’s [32] study of the impact behaviour of the mantis shrimp telson, which was found to serve as biological armour when defending against recurring energetic strikes. Zhang et al. [33] further explored the composition, fibres, and microstructural architecture of the mantis shrimp telson. The results suggest that the exceptional impact resistance of the mantis shrimp telson results from the hierarchical architecture of fibres at the micro scale.

The interior trabecular structure of beetle forewings is an example of bioinspiration for designing energy-absorbing lattice structures. Xiang and Du [34] investigated the design of the Japanese insect beetle and ladybeetle forewings. The authors explored the design of the multicell tube (MCT) lattice structures of the honeycomb walls and the wing’s circular tubes. In another study, Hao and Du [35] studied MCT structures in terms of strength and energy absorption by varying circular tube locations along the honeycomb walls. They found that certain arrangements of the circular tube and honeycomb walls provided excellent specific energy absorption. Additionally, Xiang et al. [36] designed MCT lattices with polygonal order instead of circular tubes similar to the ladybeetle internal geometry for the fabrication of lightweight lattice structures with controlled energy absorption. Other studies have explored bioinspired multicell tubes (BMTs) based on the forewing of the ladybeetle, which has shown excellent energy absorption capabilities comparable to bitubular structures [37,38,39]. Studies have shown that the cross-sectional shape and inner wall thickness of BMTs impact their energy absorption. Zhang et al. [40] investigated the effect of circular tube location and cross-sectional shape on BMTs with various arrangements, such as quadrilateral, hexagonal, and octagonal sections. The results showed that octagonal BMT designs exhibited excellent energy absorption compared to other shapes [41]. Zhang et al. [42] further explored the use of bionic octagonal multicell tubes. The influence of geometrical parameters, such as tube diameter, wall thickness, and number of tubes, on the energy absorption capacity of these structures was investigated. The results confirmed the potential to use octagonal BMT designs for impact-resistance applications.

Honeycomb cellular structures are shown to have excellent stiffness and specific strength. However, the load-carrying capacity of these designs is constrained by the anisotropic properties of these structures, which can lead to undesired buckling or bending during deformation when subjected to uniaxial loads. Localised stress concentrations can also develop at tube junctions inside the honeycomb structure, potentially leading to material failure. To address this issue, various studies have explored the use of minimum surface-like topologies in lattice material or structural designs [43,44]. The aim was to reduce the effect of anisotropy and, as a result, the risk of buckling and bending under uniaxial load. It has been demonstrated that altering the lattice’s relative density, topology, or cell size can enhance the material’s load-carrying capability. By overcoming these constraints, honeycomb cellular structures can have a wider range of applications, including protective gear, energy absorption systems, and lightweight structures [45,46]. Triply periodic minimum surface (TPMS) structures are mathematical models made up of non-intersecting and minimum surfaces. Notably, TPMS structures show topological similarities with biological structures, such as sea urchin skeletal plates, making them suitable for bioinspired lattice construction. They are also characterised by their ability to reduce surface area while retaining low curvature, with no sharp corners that may cause localised stress concentration and potential failures [47,48,49].

As discussed above, the literature has offered insights into the application of bioinspired structures, such as in the design of bio-inspired lattice structures. However, grading the topological design of these structures to improve mechanical performance under compression and impact loading circumstances has not been the subject of any previous research. The lack of literature on grading bioinspired lattice structure designs is evidently due to the need to implement multidisciplinary tasks, including topology optimisation, bioinspired design, and adapting manufacturing technologies to achieve the required intricate design. These challenges, together with difficulties in designing, simulation, and fabrication, have discouraged extensive studies on this topic. Therefore, this study presents a novel approach to the construction of functionally graded, bioinspired, and uniform lattice structures with minimum surface features, as observed in the mantis shrimp and the forewings of beetles. Compressive strength and impact characterisations were carried out numerically and experimentally to identify the ideal arrangement of the designed structures for improved strength and energy absorption.

## 2. Materials and Methods

### 2.1. Implicit Design Strategies

**Figure 1:**
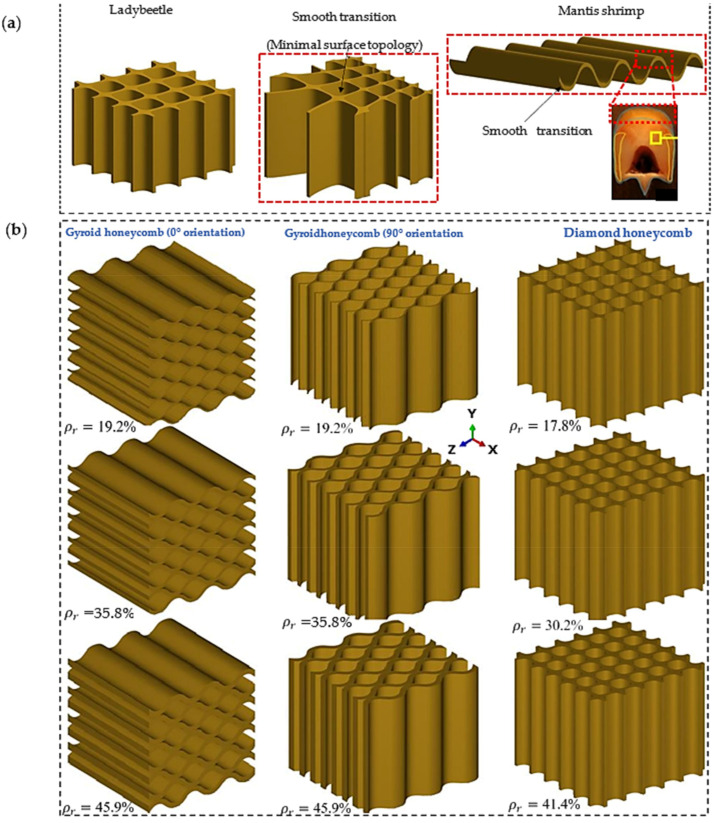
(**a**) Ladybeetle forewing microstructure [9,34], Mantis shrimp structure that inspired lattice designs [50], and specific features that inspired lattice designs. (**b**) Uniform minimal surface diamond and gyroid honeycomb lattices at different orientations.

Uniform and functionally graded lattice structures with minimal surface honeycomb lattices are designed and shown in Figure 1a. They were inspired by the microstructure of a beetle’s forewing, the topology of a Mantis shrimp shell, and a dactyl club [9,34,50]. In addition, the mathematical equations used to generate a TPMS lattice were the level-set approximation equations of the Schwartz diamond (*D*) and Schoen gyroid (*G*), as given in Equations (1) and (2) [47].

*D* = *cos*(*x*) ∗ *cos*(*y*) ∗ *cos*(*z*) − *sin*(*x*) ∗ *sin*(*y*) ∗ *sin*(*z*) = 0 (1)

*G* = *sin*(*x*) *cos*(*y*) ∗ *sin*(*x*) *cos*(*y*) ∗ *sin*(*x*) *cos*(*y*) = 0 (2)

To design bioinspired structures with minimal surface lattices, the equations’ variables are defined as *x* = 2*παX*, *y* = 2*πβY*, and *z* = 2*πγZ*, where *X*, *Y*, and *Z* represent coordinates inside the rectangular domain. The unit-cell tessellations are controlled by the parameters α, *β*, and *γ*, which function as periodicity terms. Structures based on minimum surface lattices are constructed using the level-set approximation formula for *D.* A MATLAB, 2023b algorithm incorporates the constraint *Ds = −(D*^2^ *− t*^2^*)* to determine the lattice structure’s relative density or thickness [47]. For the *D* and *G* equations, the values of ‘*t*’ are defined as $\left(\frac{1}{120}\right)\rho_{r}$ and $\left(\frac{1}{65}\right)\rho_{r}$, respectively, where $\rho_{r}$ is the relative density of the lattice structure. To design honeycomb bioinspired structures, it is important to constrain the evolution of the isosurface in a specific direction. As shown in Figure 1b, the developed MATLAB code was employed to design a uniform gyroid honeycomb (GHu) in the 0° and 90° orientations. For a GHu in the 0° orientation, the terms α and *β* are equal to zero, whereas for a GHu in the 90° orientation, *β* is equal to zero.

The diamond honeycomb (DHcs) lattice structure was created by grading the uniform (Dhu) lattice cell size in a single direction. The diamond-like form and cell size variation of the beetle’s forewing internal microstructure illustrated in Figure 1a inspired this design. The cell size of the lattice is graded in the z-direction using a differential equation solution [38]. The steps to the equations’ solutions can be explained as follows:

- Define the constants using Equations (3)–(5).
  *α* = *k *· *Z* + *a* (3)
  *β* = *k* · *Z* + *a* (4)
  *γ* = (*k*/2) · *Z* + *a* + *b*/*Z* (5)
- It was given that:
  *a* = −*Z_min_ k* + 1 and *b* = 0.5 *k* (*Z_min_*)^2^
- *K* can be calculated as *k* = (*m* − 1)/(*Z_max_* − *Z_min_*).
- *K* can be calculated as *k* = (*m* − 1)/(*Z_max_* − *Z_min_*).
- With given values for *Z_min_*, *Z_max_*, *m*, calculate *k* using the provided formula, then substitute *k* into the equations for *a* and *b* to find their values. Following that, use these values of *a* and *b* to compute α, β, and γ for each point in the lattice structure.
- For suggested values for *m*, (e.g., *m* = 1.5 or 2) repeat the calculation process to obtain corresponding values for *a*, *b*, *α* and *β* and *γ*.

**Figure 2:**
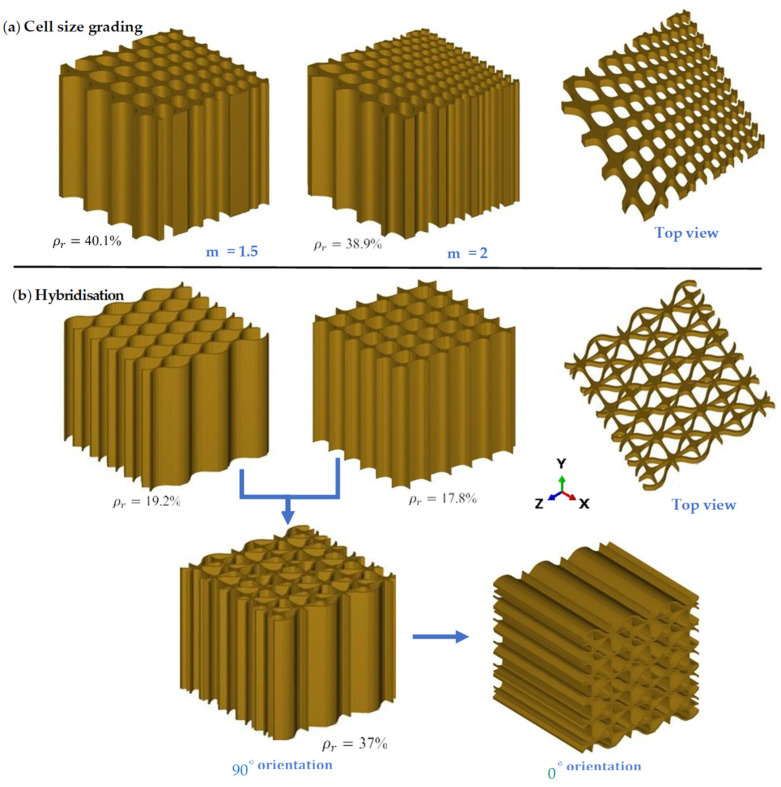
(**a**) Cell-size graded DH lattice structure at two different cell-size multipliers, and (**b**) hybrid lattice structure presented at two different orientations.

In this study, two values, *m* = 1.5 and *m* = 2, were chosen to compare the effect of the DHcs lattice structure to the DHu lattice structure. The values of the cell-size multiplier were chosen based on previous research carried out by Al-Ketan et al. [47]. Lattice hybridisation is a process that combines two or more separate lattice structures to form a new hybrid structure, as seen in Figure 2b [51]. Two separate mono-lattices are merged in this structure to form a new hybrid structure. The aim is to allow the hybrid structure to maintain a greater density microstructure per unit volume, resulting in enhanced local densification and higher stress resistance before reaching maximum densification strain during loading. This, in turn, increases the hybrid structure’s energy absorption capacity, making it more effective in mitigating the effect of bending under loading [52,53].

### 2.2. Computational Modelling

#### 2.2.1. Material Modelling

Polylactic acid (PLA) material was considered in this study as one of the organic-based thermoplastic polymers that can be prepared from corn-based starch or sugar, which makes it an available and safe-to-use material. It is a popular material used in many applications because of its biocompatibility, biodegradability, and sustainability. PLA material typically shows ductile deformation behaviour at room temperature. However, although not as ductile as metals and metal alloys, it can undergo plastic deformation before it fails, allowing it to absorb energy during mechanical loading [54]. When modelling PLA in FEA simulations, it is important to select a material model that accurately represents its real deformation response. The Johnson–Cook model is one of the most widely used constitutive models to represent mechanical behaviour under various loading conditions. To model the elastic–plastic region of the PLA polymer under quasi-static loading, the first part of the Johnson–Cook model was used to determine the relationship between the equivalent flow stress (*σ_eq_*) and parameters such as the yield strength (*A*), strain hardening modulus (*B*), strain hardening exponent *(n*), strain rate parameter (*ε*), and strain rate coefficient (*C*); see Equation (6). For impact loading, the dynamic part of the Johnson–Cook model was then activated in the FEA model to represent the material’s dynamic behaviour during high-strain-rate loading, such as impact or crash.

(6) $$\sigma_{eq}=\left(A+B\varepsilon_{p}^{n}\right)\left(1+C\;ln{\dot{\varepsilon }}^{*}\right)$$

where, *ε_p_* represents the plastic strain, ${\dot{\varepsilon }}^{*}$ is the normalized strain rate, $\dot{\varepsilon }$ is the strain rate, and *ε*_0_ = 0.001/s is the reference strain rate. The Johnson–Cook damage model in Equation (7) shows the fracture plastic strain $\varepsilon_{f}^{p}$, which depends on the stress triaxiality ratio η. However, for the strain rates and relative densities examined in this study, it was assumed that heat has negligible effects on the stress response of sheet-based TPMS lattice materials. The moderate strain rates prevent significant thermomechanical coupling. Additionally, the low relative densities limit heat transfer due to the high surface area-to-volume ratio. Therefore, the thermal softening term in the Johnson–Cook model can be neglected while still providing an accurate representation of the lattices’ mechanical behaviour.

(7) $$\varepsilon_{f}^{p}=\left(D_{1}+D_{2}e^{D_{3}\eta }\right)\left(1+D_{4}ln{\dot{\varepsilon }}^{*}\right)$$

where η is defined as the ratio of mean stress (*σ_m_*) to the equivalent Mises stress (*σ_eq_*), and *D*_1_, *D*_2_, *D*_3_, and *D*_4_ are the parameters associated with damage. A damage initiation law was used to characterise how the stiffness and strength of the base material degrade after meeting the damage initiation condition.

(8) $$D=\int \frac{d\varepsilon_{p}}{\varepsilon_{f}^{p}\left(\eta ,{\dot{\varepsilon }}^{*}\right)}$$

where *D* is cumulative damage and failure occurs as it approaches a value of 1. The material properties of the PLA used in this study are provided in Table 1. It is assumed that the damage evolution initiates at a displacement to failure of 0.38. It is important to note that these values were derived through parametric studies and by comparing numerical results in quasi-static compression with experimental observations.

#### 2.2.2. Computational Process

**Figure 3:**
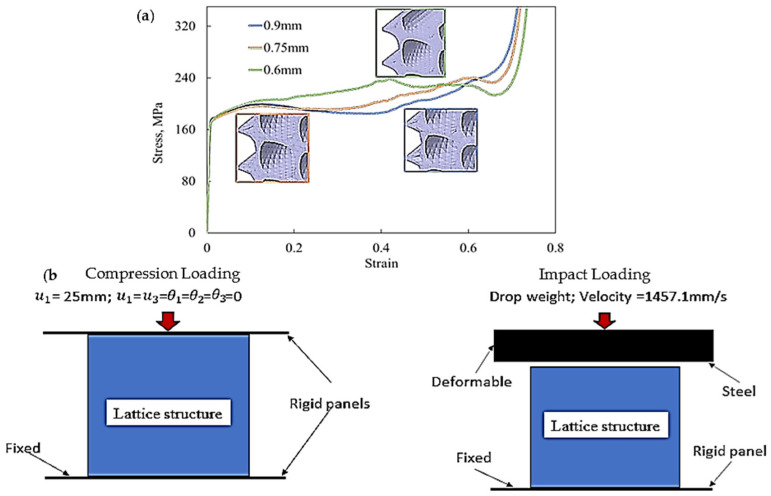
(**a**) Plot comparing the effect of element size on the compressive stress–strain behaviour of DHu and (**b**) a schematic representation of mechanical loading and boundary conditions.

Different lattice designs were created and imported into Altair@ Hypermesh^®^ software in standard tessellation language (STL) format. The model was then meshed using second-order quadratic tetrahedral elements. The mesh sensitivity analysis was then used to evaluate the effect of element sizes on the simulation results and to determine the appropriate element sizes for the FEA simulation. This was achieved by varying the elements’ sizes and evaluating their effects on the simulations’ accuracies and computing times. Table 2 displays the maximum plastic strain and computational time, where the element size was selected between 0.9 mm and 0.45 mm. It can be noted that the maximum plastic strain increased by just 0.01 when the element size was reduced from 0.6 mm to 0.45 mm, although the computation time was nearly quadrupled. In addition, the deformed mesh displayed severe deformation and a poor aspect ratio when the element size was greater than 0.6 mm (i.e., 0.9 mm and 0.75 mm), as demonstrated in Figure 3a. As a result, an element size of 0.6 mm was selected. Figure 3a shows the full stress–strain diagram at element sizes of 0.9 mm, 0.75 mm, and 0.6 mm to demonstrate the mesh distortion found when element sizes of 0.9 mm and 0.75 mm were used. The stress–strain diagram at an element size of 0.45 mm was not included, as it quite overlaps with that at 0.6 mm. For these reasons, an element size of 0.6 mm was chosen. Figure 3a shows the stress–strain diagram when using element sizes of 0.9 mm, 0.75 mm, and 0.6 mm to showcase the mesh irregularities when using element sizes of 0.9 mm and 0.75 mm. On the other hand, the stress–strain diagram of an element size of 0.45 mm was not included in the figure, as it overlaps with that of 0.6 mm.

The mesh was then exported to Abaqus/CAE 2017 (Dassault Systemes, Velizy-Vilacoublau, France) for simulation. Two rigid panels with dimensions of 50 mm × 50 mm were created for the compression simulation to act as top and bottom loading parts. PLA material properties, shown in Table 1, and the boundary conditions were assigned to the mesh, as shown in Figure 3b. During compression loading, the rigid panels would come into contact with the lattice, and the lattice structure itself would exhibit self-contact during various stages of densification. Therefore, hard contact with a friction coefficient of 0.4 was applied between the panels and the lattice structures. Abaqus/Explicit was employed to study the quasi-static compression with a time step of 0.01 s for the nonlinear and dynamic responses. The effect of inertia was found to reduce when the time increment became small, which enabled the FEA simulation to carry out the compression behaviour investigation without the need to study the dynamic analysis. 

Smooth stepping was activated to minimise the influence of inertia. It is important to emphasise that the small time increment used would not produce a dynamic or time-dependent response from the lattice. This is because in the quasi-static compression simulations, the dynamic term of the Johnson–Cook models, C, is equal to zero. On the other hand, for the impact loading, a deformable block was produced. However, a time step of 0.02 s was used to allow for the complete absorption of the impactor’s kinetic energy. In this scenario, the dynamic response of the Johnson–Cook models was activated.

#### 2.2.3. 3D Printing

**Figure 4:**
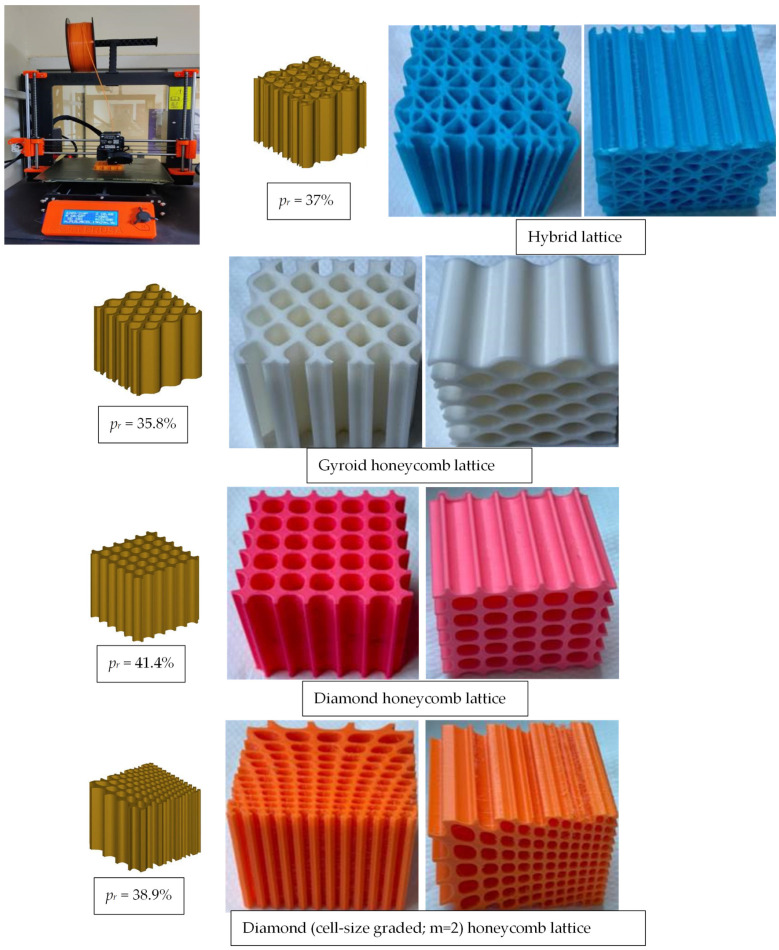
Image showing 3D printing of digital designs through the FDM process and 3D printed samples.

The various CAD models and experimental fabricated lattice structures built using 3D printing from PLA materials are shown in Figure 4, each with an overall dimension of 60 mm × 60 mm × 60 mm. The 3D printing process began with the loading of a digital STL file into the 3D printer, which was then 3D printed layer by layer according to the model. Table 3 contains the detailed process parameters for the 3D printing process, and the weights of the fabricated cellular structures are listed in Table 4.

#### 2.2.4. Compression Testing

**Figure 5:**
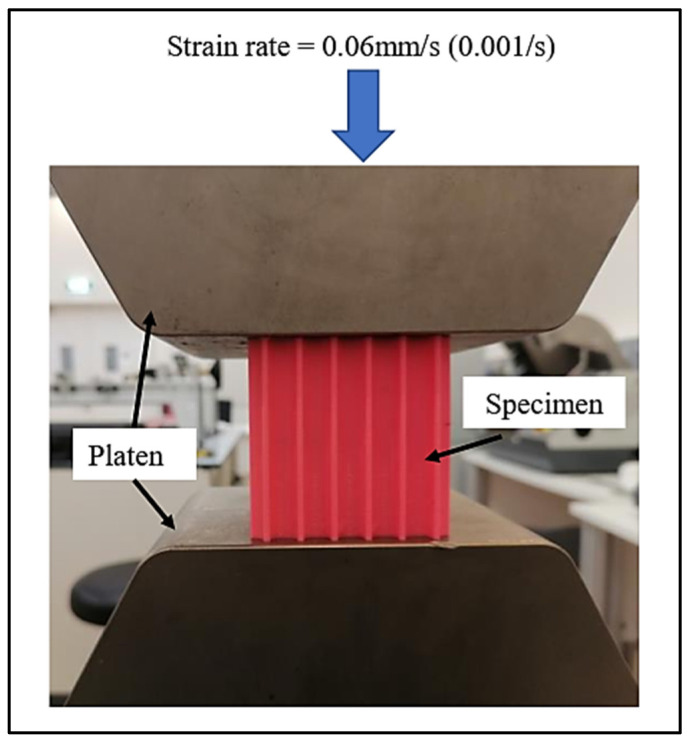
An image showing the sample placed between the platens in a compression test.

Figure 5 depicts the compressive testing performed on one of the lattice systems illustrated in Figure 4. The goal was to investigate the stress–strain response of various lattice designs until they reached maximum deformation. The strain rate used in the investigation was 0.06 mm/s. Stress was calculated by dividing the force applied to the specimen by its cross-sectional area (60 mm × 60 mm), while strain was calculated by dividing the displacement by the gauge length (also 60 mm).

## 3. Results and Discussions

### 3.1. Effect of Relative Density

The mechanical qualities of lattice systems are related to their geometrical shape, with a particular emphasis on relative density. In principle, increasing a lattice structure’s relative density should result in enhanced mechanical properties. However, the degree of improvement is significantly influenced by the shape of the lattice structure. Through a series of numerical simulations, this study explored the effects of the relative density of bioinspired shapes (DHu and GHu lattice designs) on their compressive elastic modulus (E), peak strength (S), and energy absorption (EA).

**Figure 6:**
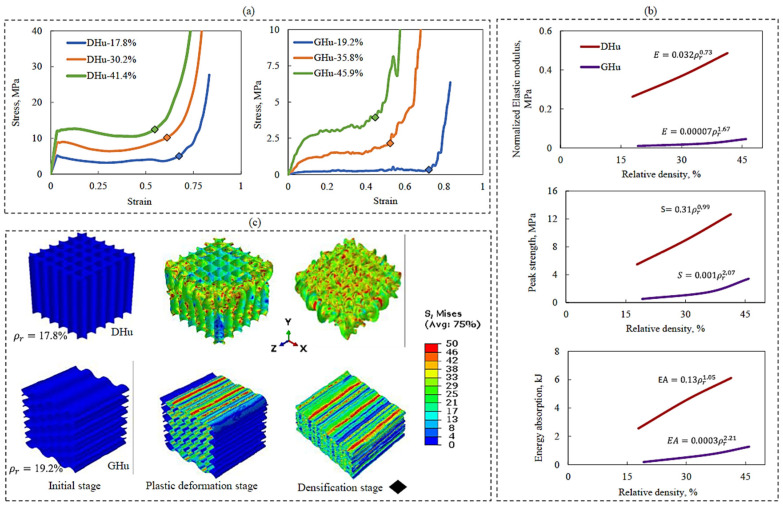
Effect of lattice relative density on the mechanical properties of DHu and GHu lattice structures; (**a**) quasi-static compressive stress–strain response, (**b**) compressive properties, (**c**) Mises stress contours showing stage-wise deformation patterns.

Figure 6a–c shows the compressive stress–strain behaviour, compressive properties, and Mises stress contour plots for the DHu and GHu lattices, respectively. As shown in Figure 6a, compressive stress–strain responses of lattices of varied designs and relative densities display comparable tendencies. This behaviour often begins with a linear elastic phase, progresses to a plateau, and ends with a constant increase in compressive stress magnitude. The stress response deviates from linearity throughout the plateau phase, displaying a gradual variation that occurs until the point of densification is attained, as evidenced by the diamond-shaped points. This variation is due to the deformation of the first layer of unit cells and their subsequent densification in the following layer. This phenomenon is less evident in the DHu lattice than in the GHu lattice, owing to differences in unit-cell orientation. The DHu lattice has unit cells that are parallel to the compression loading direction, whereas the GHu lattice has unit cells that are perpendicular to the loading direction. 

Figure 6b shows that the DHu lattice design consistently outperforms the GHu design in terms of elastic modulus, peak strength, and energy absorption properties throughout a wide range of relative densities. This advantage is mostly due to the orientation of the unit cells, as previously explained. The rise in quasi-static compressive characteristics for the DHu lattice is essentially linear, but the increase for the GHu lattice is exponential. Furthermore, the Mises stress contour plots in Figure 6c explain the deformation patterns of the two bioinspired lattice architectures. Deformation patterns in the DHu lattice are mostly bending dominated, whereas deformation patterns in the GHu lattice are stretching dominated. These diverse deformation properties highlight the importance of lattice topology in influencing mechanical responses and are in agreement with the literature [52]. As a result, it was important to investigate changing the orientation of the unit cells to 90° in order to improve the mechanical characteristics of the GHu lattice. The results will be discussed in depth in the next section.

### 3.2. Effect of Bioinspired Topology

**Figure 7:**
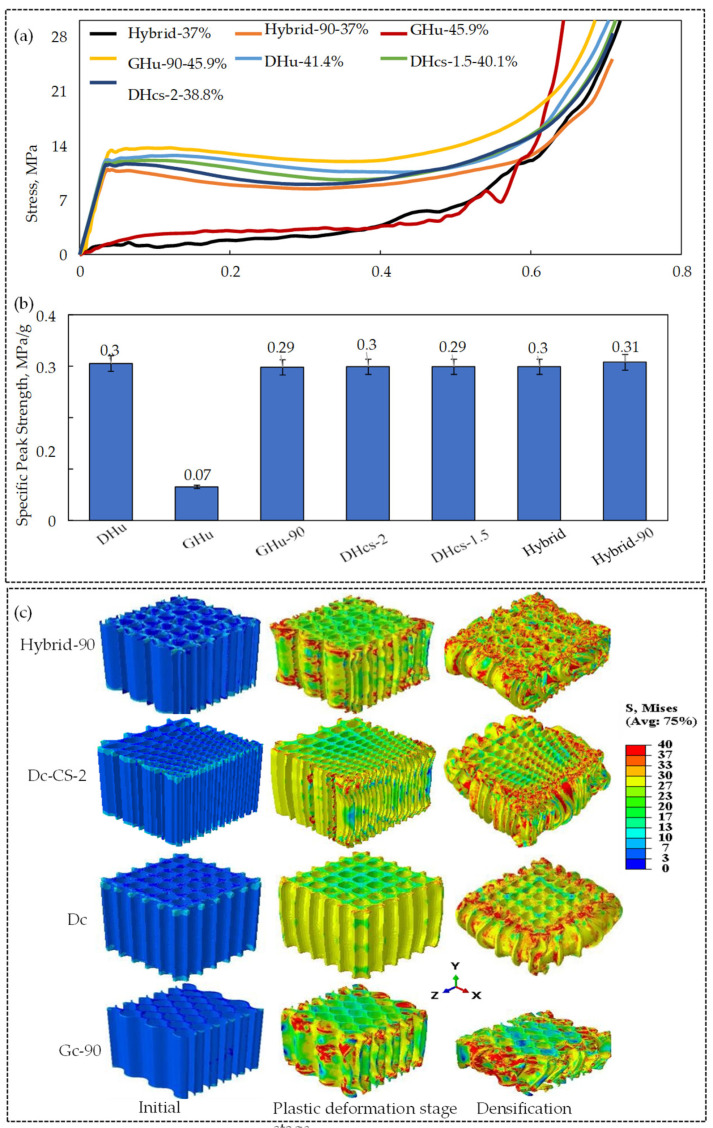
Illustration of the influence of lattice topology on the mechanical performance at different stages of compression; (**a**) Quasi-static compressive stress–strain diagram, (**b**) Specific peak strength, and (**c**) Mises stress contour plots of the deformation behaviour.

To study the effect of the bioinspired topology, a fixed lattice relative density was maintained, while the influence of other factors, such as cell size gradation, hybridisation, and unit-cell orientation, on compressive specific peak strength was investigated. It is worth noting that the maximum strength is calculated as the maximum stress of the lattice right before the point of final densification, and that the specific strength is the division between the maximum strength by the lattice relative density. Figure 7a shows stress–strain diagrams for various lattice topologies, including the DHu lattice, GHu lattice, GHu-90 (GHu lattice at a 90° orientation), DHcs-1.5 (cell size graded with a multiplier of 1.5) lattice, DHcs-2 (cell size graded with a multiplier of 2), hybrid (0° orientation) lattice, and hybrid-90 (90° orientation) lattice. Except for the GHu and hybrid lattice designs, the figures show that there are no noticeable differences in the stress–strain response across the different lattice topologies. The yield strength of the GHu and hybrid lattice designs, on the other hand, was much lower, allowing them to approach the plateau phase at much lower stress levels. Notably, when orientated at 90°, the stress–strain response of GHu and hybrid lattices increased significantly. The effect of lattice topology on compressive peak strength and deformation behaviour, as shown in Figure 7b,c, appears to be negligible. Except for the GHu design, all the lattice structures displayed a particular peak strength of roughly 0.3 MPa (as shown in Figure 7b). Furthermore, the Mises stress (Figure 7c) revealed no significant changes in deformation behaviour across different lattice designs during the plastic deformation or densification phases. Following that, the numerical results shown in Figure 7 were confirmed by experiments. The GHu-90, DHu-90, hybrid-90, and DHcs-2 lattice designs were chosen specifically because they had shown considerably enhanced performance in quasi-static compressive stress throughout our analytical analysis.

**Figure 8:**
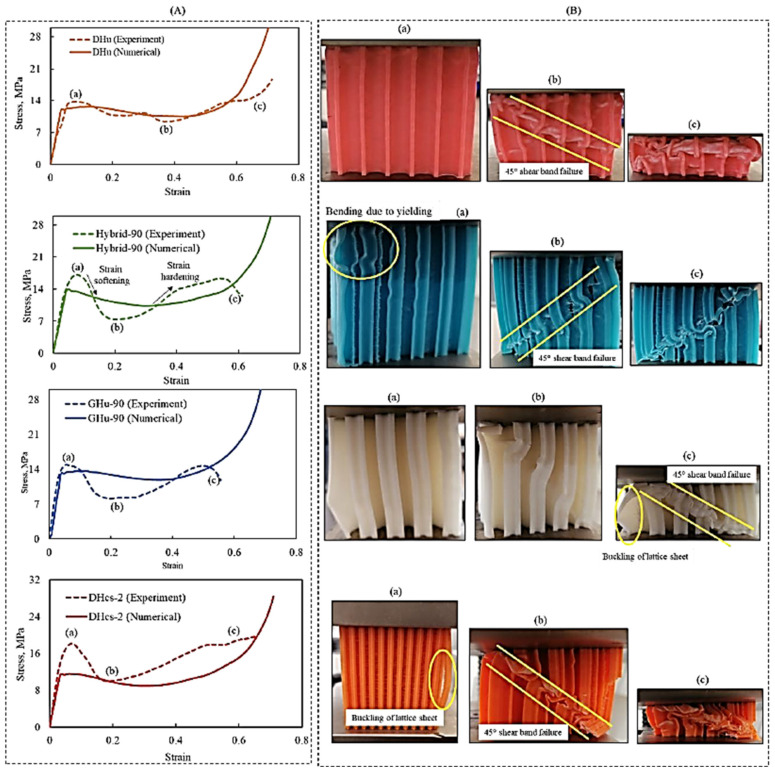
(**A**) Experimental and numerical quasi-static compressive stress–strain responses, (**B**) experimental deformation patterns for DHu, GHu-90, hybrid-90 and DHcs-2 3D-printed samples.

Figure 8A compares the simulation and experimental results of the compressive tests of four distinct lattice designs, DHu, GHu-90, hybrid-90, and DHcs-2. The same figure depicts the experimental stress–strain and numerical simulation for each lattice design. On the other hand, Figure 8B shows the deformation of the 3D-printed lattices during the compressive test. The experimental and computational findings were relatively consistent, demonstrating the numerical model’s ability to capture the major aspects of lattice behaviour during compression loading. As shown in Figure 8A,B, the stress–strain response began with an elastic area, followed by a plastic region, where the material began to deform irreversibly. Then, plastic deformation occurred until densification of the lattice structure at point (a), the stage where the lattice cells collapsed, and the structure became more compact. The sample deformed through a diagonal shear band after the yield point, resulting in a post-yield softening behaviour until reaching a minimum stress value at point (b). This stress reduction could also be due to lattice sheet buckling, which is a phenomenon where the lattice walls bend and lose their load-bearing capacity. This caused the sample to lose a significant amount of its compression resistance and consequently led to a drop in the experimental stress–strain response. The stress then increased again as the densification progressed, and the material became more resistant to further deformation. After the initial deformation, the sample attained a constant stress level, with successive layers bending along the shear band until point (c), the densification phase. The stress level changed based on the lattice form, influencing stress–strain behaviour. Distinct lattice architectures have distinct mechanical reactions under dynamic loading, and lattice mismatches can affect a material’s stress rupture life. When the material was under strain, the stress varied, lowering when a layer of cells fractured or collapsed and rebounding when the collapsed layer compacted and passed the load to the next layer. A 45° shear-band failure disturbed the unit-cells’ load-bearing capability, lowering the number of cells that withstood the applied strain. This failure mechanism agrees with the softening-like profiles observed in some lattice materials with low strain rates [40,45,47].

**Figure 9:**
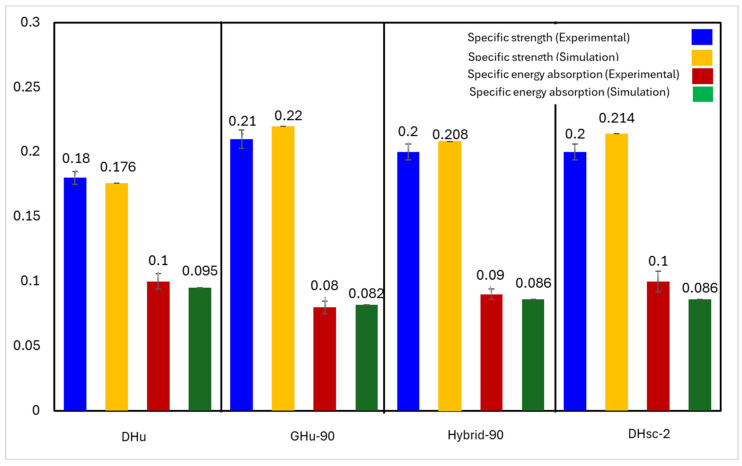
A graphical representation of the relative strength and energy absorption (in kJ/g) of the 3D-printed specimens under testing.

The quasi-static compressive test on four distinct bioinspired PLA lattice structures (DHu, GHu-90, hybrid-90, and DHcs-2) revealed that their specific strength and specific energy absorption were comparable, with mean values of around 0.2 MPa/g and 0.09 kJ/g, respectively. See Figure 9. A comparison of the experimental and simulation results for the specific strength and the specific energy absorption of the four lattice structure designs shows little variation between them but within an acceptable range. While some lattice designs show higher experimental specific strength when compared to the simulation results, other designs show the opposite behaviour. These results agree with theoretical expectations based on the lattice’s relative density and Young’s modulus. Among the four designs, the GHu-90 lattice had the best specific strength, while the DHcs-2 cellular structure had the highest specific energy absorption. This might be attributed to the lattices’ various geometrical properties and deformation processes. To assess the performance of the bioinspired lattices, they were compared to relevant results from the literature. In an earlier work by Seek et al., the performance of traditional PLA lattice structures, such as simple cubic (SC), honeycomb (HC), body-centred cubic (BCC), and PeckGy80 (PG80), was investigated [55]. The relative density was in the range of 0.16 to 0.38, which is similar to the range of the bioinspired lattices (from 0.15 to 0.40).

On the other hand, the specific energy absorption of traditional lattice structures was lower than that of bioinspired lattices, with a range of 0.08 to 0.87 kJ/kg. The developed bioinspired lattice structures absorbed energy of about 90 kJ/kg, which is 100 to 1000 times more than the traditional designs. This suggests that the bioinspired lattice design replicated from a beetle’s forewing significantly improved the crush energy absorption of the PLA lattices. In a study by Alizadeh-Osgouei et al. [56], PLA gyroid structures with a porosity level of 73% (relative density of 27%) were manufactured via FDM. Dense PLA samples were also printed in this study. The average compressive strength of the gyroid structures was about 4 MPa, while that of the dense PLA was about 56 MPa. In the current study, and as shown in Figure 8A, the compressive strength of the four bioinspired lattices produced ranged from 14 to 18 MPa, which is about four times higher than that in the work by Alizadeh-Osgouei et al., while their relative densities were at most doubled. The bioinspired lattices also achieved a strength of about one-third that of the solid material. In another study of the mechanical properties of FDM-printed PLA diamond honeycomb structures, which was carried out by Dwivedi et al. [57], the compressive properties were also assessed. For lattices whose relative densities varied from 10 to 50%, the ranges of compressive strength and specific energy absorption were from 0.009 to 0.024 MPa and from 4 to 6 J/kg, respectively. When compared to the corresponding properties of the bioinspired lattices in the present work, it can be clearly demonstrated that the compressive strengths and energy absorptions of the bioinspired lattices were higher by about three and four orders of magnitude, respectively, compared to the lattice produced in the study by Dwivedi et al. As a result, bioinspired lattices may be better suited for compression loading applications than traditional lattices.

### 3.3. Drop-Weight Impact Behaviour

**Figure 10:**
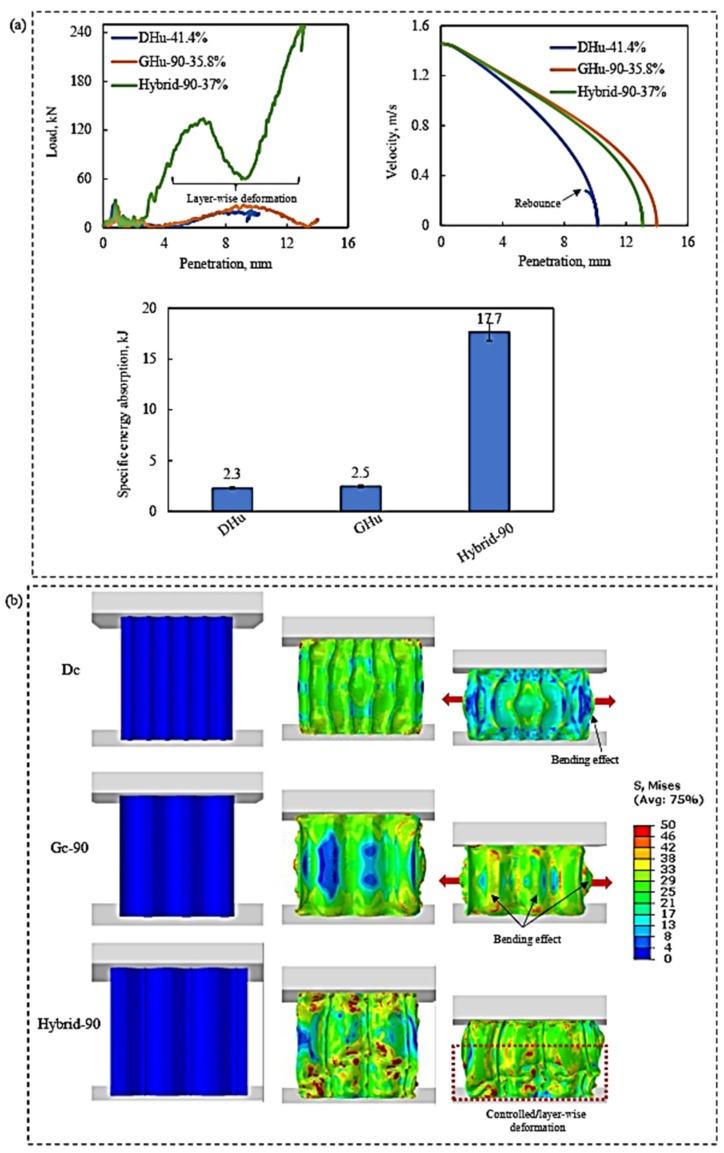
(**a**) The penetration depth and speed vary with the applied load, and a bar chart comparing how different types of lattices (DHu, GHu-90, and hybrid-90) respond to a falling weight. (**b**) The distribution of stress in the lattices when they are impacted by a drop-weight.

Based on their performances under quasi-static compression stress, this section gives a computational examination of the drop-weight impact behaviour of three lattice structures, DHu, GHu-90, and hybrid-90. The goal was to investigate how impact loading influences the strain rate and impact response of a lattice construction. Figure 10a depicts the load-penetration and velocity-penetration curves of the three lattice constructions subjected to the drop-weight impact test. A bar graph depicting the specific energy absorption of several lattice patterns throughout the test is shown in the same figure. Figure 10b shows the Mises stress contour diagrams that depict the key deformation characteristics of the lattices under loading.

In the load-penetration diagram, which quantifies the force required to pierce a material, the hybrid-90 lattice outperformed other structures. The velocity-penetration diagram also reveals a large drop in the impactor’s kinetic energy (with a negative parabolic curve) as the penetration distance rises. This indicates that the hybrid-90 lattice absorbed more energy from the collision, thus reducing structural damage. The DHu lattice had the lowest penetration, as seen in the figure, although this does not always mean superior performance. The load-penetration and velocity-penetration graphs demonstrate that although having less penetration, the DHu lattice exhibited less resistance to impact force than the hybrid-90 lattice. 

This disparity might be explained by their deformation behaviours. As seen in Figure 10b, both the GHu-90 and DHu lattices had significant bending-based failures, reducing their capacity to withstand the load. When a material bends or buckles under stress, it loses strength and stiffness. The hybrid-90 lattice, on the other hand, exhibited regulated layer-by-layer deformation behaviour, as seen by the bar chart in Figure 10a, which was the primary reason for its improved drop-weight impact performance. Because of the tight arrangement of the unit cells, the lattice has a stronger tendency to locally densify, which might explain this deformation behaviour. Local densification is a phenomenon in which the material becomes more compact and denser during compression, aiding in the dissipation of bending energy and allowing unit cells to provide load resistance. In this situation, local densification would benefit the lattice’s impact performance [45].

## 4. Conclusions

This study aimed to create bioinspired lattices that mimic the microstructure of a beetle’s forewing, a mantis shrimp’s shell, and a mantis shrimp’s dactyl club. The mechanical properties of these lattices were evaluated by FEA and experimental quasi-static compression tests that considered the impact of cell size gradation and hybridisation on lattices. The results of this study showed that the compressive elastic modulus, peak strength, and energy absorption of the lattice increased significantly with relative density, and the diamond honeycomb (DH) design outperformed the gyroid honeycomb (GH) structure in the quasi-static compressive test. Furthermore, the orientation of unit cells with respect to the loading direction had a significant impact on the mechanical behaviour of lattice structures under uniaxial loading conditions, with lattices with parallel unit cells exhibiting much higher compressive performance, up to three times higher than those with the same relative density. Furthermore, grading the cell size and hybridising the lattices altered the deformation behaviour of DHu lattices (a hybrid of DH and GH), which minimised the development of shear-band in the lattice. Bioinspired lattices had a specific energy absorption that was 100 to 1000 times greater than traditional lattices made of the same PLA material. The bending-dominated deformation, on the other hand, was minimised by compacting the lattice unit cells, which enabled the lattice to collapse in a controlled manner under impact loads, eventually increasing the specific energy absorption of the lattice by more than 200%. The study’s results can have a wide range of applications where lightweight, energy-absorbing structures are desired to improve the safety and performance of lightweight structures.

## Figures and Tables

**Table 1:** PLA elastic properties and calibrated Johnson–Cook model parameters.

*E* [MPa]	*υ*	*A* [MPa]	*B* [MPa]	*n*	*C*	*D*_1_	*D*_2_	*D*_3_	*D*_4_
830	0.37	28	0.68	7.709	0.05	0.04	2.46	0.96	0.01

**Table 2:** Mesh sensitivity of the model.

Element size (mm)	0.9	0.75	0.6	0.45
Max plastic strain	0.64	0.67	0.7	0.71
Computational time (minutes)	15	28	47	92

**Table 3:** FDM printing parameters.

Layer Thickness (mm)	Flow Rate (mm^3^/s)	Printing Speed (mm/s)	Temperature of the Nozzle (°C)	Temperature of the Build Plate (°C)	Retraction Distance (mm)
0.2	100	120	120	60	5

**Table 4:** Mass of different lattice structures of different designs.

		Mass (g)		
Lattice design	Gyroid honeycomb	Diamond honeycomb	Diamond cell size graded honeycomb	Hybrid honeycomb
	73	74	89	86

## Data Availability

The data presented in this study are available on request from the corresponding author.
